# Assessment of Patient Matters in Healthcare Facilities

**DOI:** 10.3390/healthcare12030325

**Published:** 2024-01-26

**Authors:** Flaviu Moldovan, Liviu Moldovan

**Affiliations:** 1 Orthopedics—Traumatology Department, Faculty of Medicine, “George Emil Palade” University of Medicine, Pharmacy, Science, and Technology of Targu Mures, 540142 Targu Mures, Romania; 2 Faculty of Engineering and Information Technology, “George Emil Palade” University of Medicine, Pharmacy, Science, and Technology of Targu Mures, 540142 Targu Mures, Romania; liviu.moldovan@umfst.ro

**Keywords:** patient matters, sustainability, orthopedics, reference framework, healthcare facility, assessment

## Abstract

Background and Objectives: Ensuring the sustainability of healthcare facilities requires the evaluation of patient matters with appropriate methods and tools. The objective of this research is to develop a new tool for assessing patient matters as a component of social responsibility requirements that contribute to the sustainability of healthcare facilities. Materials and Methods: We carried out an analytical observational study in which, starting from the domains of the reference framework for the sustainability of health facilities (economic, environmental, social, provision of sustainable medical care services and management processes), we designed indicators that describe patient matters. To achieve this, we extracted from the scientific literature the most recent data and aspects related to patient matters that have been reported by representative hospitals from all over the world. These were organized into the four sequences of the quality cycle. We designed the method of evaluating the indicators based on the information couple achievement degree-importance of the indicator. In the experimental part of the study, we validated the indicators for the evaluation of patient matters and the evaluation method at an emergency hospital with an orthopedic profile. Results: We developed the patient matters indicator matrix, the content of the 8 indicators that make it up, questions for the evaluation of the indicators, and the evaluation grids of the indicators. They describe five levels for each variable of the achievement degree-importance couple. The practical testing of the indicators at the emergency hospital allowed the calculation of sustainability indicators and the development of a prioritization matrix for improvement measures. Conclusions: Indicators designed in this research cover social responsibility requirements that describe patient matters. They are compatible and can be used by health facilities along with other implemented national and international requirements. Their added value consists in promoting social responsibility and sustainable development of healthcare facilities.

## 1. Introduction

Governments and funders have included sustainability among the fundamental criteria for evaluating public healthcare institutions. This is due to the urgency of social and environmental issues that dominate contemporary society. From these considerations, the need to ensure medical assistance with added value for the patient, which constitutes sustainable care, was outlined [1]. The sustainability of healthcare programs is directly related to the quality and accessibility of healthcare. Therefore, it is necessary to identify, periodically evaluate, and continuously improve the defining aspects of patient matters.

In the specialized medical literature, patient matters are studied by researchers from different perspectives, which allows an exhaustive identification of the defining aspects. These refer to the models of relationship with the medical staff, the development of patient trust, the provision of medical services, self-care programs, chronic conditions, ambulatory care, and patient safety. All of these patient matters are integrated into the sustainability of healthcare programs. In addition, patient matters contribute to the evaluation of the performance of hospitals through criteria related to the experience they encounter. Thus, the experience of patients who benefited from medical insurance and electronic health management systems in large hospitals with a lower percentage of patients contributed to the registration of a high degree of satisfaction [2].

Patient matters also include relational patterns that affect the therapeutic relationship [3]. Their study requires the analysis and evaluation of the two components that make them up: the work component and the interpersonal component. In this way, an adequate dynamic of the therapist-patient interpersonal relationship is ensured, which makes a significant contribution to obtaining the therapeutic result [4]. The development of patient trust is directly generated by the professional skills and interpersonal care attributes of medical staff [5]. Confidence increases with understanding of medical staff reimbursement processes, which are most frequently overestimated by patients [6]. Another defining aspect of patient matters is the provision of self-care programs, which have the potential to improve patients’ quality of life and significantly reduce medical complications [7]. This includes elderly patients with hip fractures who are bedridden for an extended period of time. With the support of medical engineering, chronic conditions, such as those of the diabetic foot, which present risks of infections and amputations, must be improved [8]. Improvements in medical practices can be supported through four pillars identified by Vij and Beyda [9]: knowledge exchange, advocacy, research initiative and patient health education.

Also, the patient’s problems require analysis of the way in which patients are hospitalized in outpatient departments. They require continuous evaluation due to the changes that occur following the occurrence of emergency situations. For example, Asfuroğlu and Gümüşoğlu [10] found that following an earthquake, the volume of cases with trauma diagnoses in patients from outside the serviced region increased, while the number of patients with medically unconfirmed diagnoses decreased. Increasing patient safety by assessing and solving current incident reporting challenges contributes to addressing patient matters. Most often, they do not reach their potential due to erroneous processing (triage, analysis, referrals), inadequate involvement of doctors, insufficient visibility of follow-up actions, limited financial resources, ineffective incident control mechanisms, and insufficiently digitized communication systems [11]. Another aspect that requires the development of patient matters assessment tools is the treatment of complaints. When patients make complaints, there is a negative public preconception about how the complaint will be handled. This does not constitute a favorable condition for recording a positive result of the subject of complaint. The significant difference comes from the opposite expectations of patients and doctors regarding the situation complained of [12].

Each of the aspects that make up patient matters and that have been identified in the literature requires a periodic evaluation, in order to develop improvement programs that have the potential to increase the sustainability of healthcare facilities. This can be done with the support of a tool that allows a unitary evaluation of the multitude and diversity of the aspects identified. Starting from the controversies identified in the scientific literature regarding patient matters, in our study we formulated the following research questions:

(RQ1): What are the aspects that define the sustainability of patient matters?

(RQ2): What are the correct healthcare practices reported in the scientific literature and validated in practice by the internationally representative medical institutions that treat patient matters?

(RQ3): With the support of these practices, what indicators can be defined for the evaluation of patient matters?

(RQ4): How should the indicators of a new reference framework be qualitatively and numerically defined, to allow the evaluation and monitoring of progress in the implementation of patient matters?

Based on these research questions, we formulated the main objective of this research, which consists of the development of a new tool for assessing patient matters as a component of social responsibility requirements that contribute to the sustainability of healthcare facilities.

Ensuring the compatibility of this evaluation tool with the accreditation legislation, as well as with other reference frameworks currently used in hospitals, is the secondary objective of the research.

## 2. Materials and Methods

We used the following research methodology:

- Study design and selection of participants;
- Design of the reference framework domains for the assessment of patient matters;
- Data collection and analysis;
- Design of patient matters indicators and evaluation grids;
- Validation in practice of the theoretical model developed for the evaluation of patient matters at an emergency hospital.

### 2.1. Study Design and Selection of Participants

To investigate the research questions formulated in this study, we designed exploratory research of a qualitative and primary nature to collect the most relevant and recent aspects regarding patient matters. We included in the study the results of research that have been reported by representative medical units from all over the world. 

The study was conducted according to the guidelines of the Declaration of Helsinki and approved by the County Emergency Clinical Hospital of Targu Mures Ethics Committee for the practical validation of the new theoretical model.

### 2.2. The Reference Framework Domains

Patient matters are made up of a multitude of aspects that were identified in the introductory section. These have the potential to improve the quality and sustainability of healthcare facilities. For this, a reference framework based on indicators is needed, which allows their periodic evaluation, and the formulation of improvement measures. In the medical literature, there are previous quality assessment models in hospitals developed through international collaboration [13], or at the national level for the accreditation of healthcare facilities with beds [14] or in outpatients [15]. Their main omission is that they do not integrate sustainability issues related to patient matters of social responsibility.

**Figure 1:**
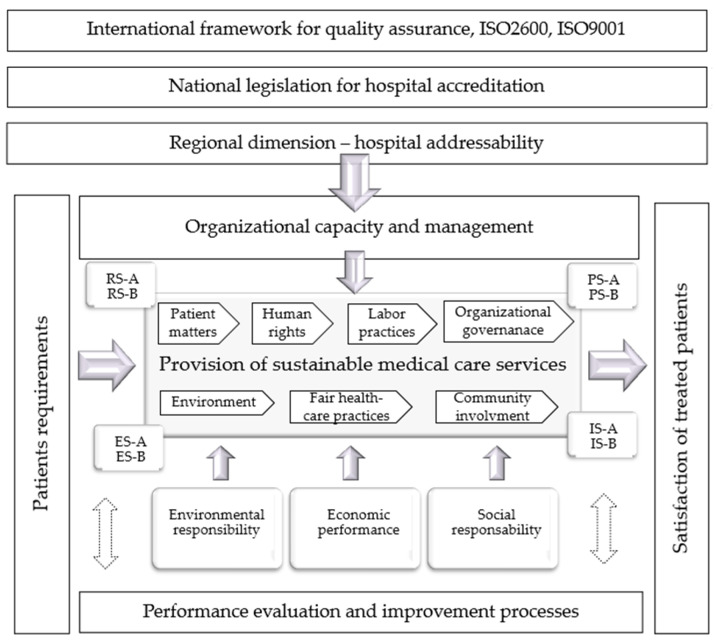
The conceptual model of the Health Sustainability (H-S) reference framework: (PS–A)–Accreditation of health system, (PS–B)–Design of patient centered medical services, (IS–A)–Medical services delivery, (IS–B)–Patient transfer, (ES–A)–Institutional outlook leaders, (ES–B)–Satisfaction degree evaluation, (RS–A)–Self-appraisal, (RS–B)–Medical services re-conception. In our model, the social area, which belongs to sustainability, is organized following the requirements of the social responsibility standard ISO 26000 [17]. By adapting the seven subfields of the standard to the particularities of the medical field we obtained: organizational governance, human rights, labor practices, environment, fair healthcare practices, patient matters, community involvement and development.

Considering this identified requirement, we established the domains of the new reference framework Health-Sustainability (H-S), so that there is support and compatibility with existing international [13] and national models [14,15] which healthcare facilities apply routinely. The subject of this research on assessment of patient matters with the support of an innovative framework continues the authors’ research from a previous article [16] in which we established the fields of the new Health-Sustainability (H-S) reference framework and based on which we investigated fair healthcare practices. For this reason, in the present study in which we investigated patient matters, we took from the previous research the domains of the new reference framework for the sustainability of health facilities (H-S): economic, environmental, social, provision of sustainable medical care services, and management processes (Figure 1) [16]. These are necessary to ensure the commitment of management that can direct medical staff to ensure the quality and sustainability of medical services.

Following the requirements of the ISO 9001 standard regarding quality assurance [18], in the next stage of the research we have organized the succession and interconnection of basic medical activities in the quality cycle. We designed two activities for each stage of the quality cycle. The healthcare service design in the PS–plan stage is composed of two activities: (PS–A)–Accreditation of health system, and (PS–B)–Design of patient centered medical services. It is followed by healthcare service provision in the IS–implementation stage, which is composed of (IS–A)–Medical services delivery and (IS–B)–Patient transfer. Next the ES–evaluation stage integrates activities (ES–A)–Institutional outlook leaders as well as (ES–B)–Satisfaction degree evaluation. In the last phase of the quality cycle the RS–review stage comprises another two activities, (RS–A)–Self-appraisal and (RS–B)–Medical services re-conception [19].

### 2.3. Data Collection and Analysis

We explored the most relevant databases—Web of Science, PubMed, EMBASE (OVID)—in which we searched for keywords: patient matters, relationship with medical staff, patient trust, performance of medical services, self-care programs, chronic conditions, outpatient, patient safety, etc. From the papers found, we extracted the most recent publications, preferably less than 10 years old, that describe aspects related to patient matters. The selection condition was that the articles present new knowledge, results of discoveries confirmed by evidence, or clinical studies. 

In this way, at the end of the research stage, we created a database with the most recent defining aspects of patient matters. The data were filtered, subjected to primary analysis, and transferred to Microsoft Office for further processing. In the case of articles that discussed the same aspect, we made comparisons and selected the contents that were in accordance with the requirements of this study. We preferred practices that allow good traceability of the analyzed activity and that present a greater degree of generality.

The methodology for processing the collected data consisted of a separate analysis for each of the basic medical activities: (PS–A)–Accreditation of health system, (PS–B)–Design of patient centered medical services, (IS–A)–Medical services delivery, (IS–B)–Patient transfer, (ES–A)–Institutional outlook leaders, (ES–B)–Satisfaction degree evaluation, (RS–A)–Self-appraisal, and (RS–B)–Medical services re-conception.

### 2.4. Evidence of Patient Matters in Healthcare Organizations

During data collection from documentation in the scientific literature and its analysis, we selected the most relevant and recent aspects regarding patient matters that have been reported by representative medical units from all over the world. We organized the database with evidence according to the sequences of the quality cycle: design of medical services, provision of medical services, evaluation of medical services and improvement of medical services, as can be seen in the following sections.

#### 2.4.1. Practices for Design of Medical Services

The public communication of information about the impact of health care provider performance and patient behavior on the quality of health care has been reported in a limited number of studies. The quality of the medical act increases with the feedback given on the clinical performance of the doctor [20], but it has different effects in the case of patients from disadvantaged social backgrounds [21]. The dissemination of information about the performance of the medical act has positive effects in patients diagnosed with acute myocardial infarction [22].

In the case of common medical conditions and orthopedic surgery, mortality statistics lead to better medical outcomes [23]. An increase in the volume of medical services [24] and improvements in outcomes, such as patient satisfaction and arthroplasty rates, [25] have been achieved. There was a small positive effect from the publication of patient outcome data on the number of patients who underwent ligamentoplasty and abnormal values with low complications for arthroscopic meniscectomies. The positive effects were observed for less than two months from the moment when the results were made known to the patients [26]. 

By combining feedback reports with quality improvement plans and continuous education of medical staff, consistent improvements in medical services have been achieved, as revealed by several studies [27].

With the support of these medical practices, we developed indicator PA6–Information regarding performance (see Table A1), which is used to evaluate medical activity (PS–A) accreditation of health system.

Promoting patient self-management is an important topic in Western health care policies. Worldwide, the integration of complementary medicine as well as alternative medicine into integrative medicine is pursued. Baars et al. [28] shows that anthroposophic medicine offers specific contributions to the promotion of patients’ self-management.

Patient and community involvement can take many forms in healthcare, but there is no single strategy or method that can be considered to reflect best practice [29]. After exploring self-management mechanisms in chronic diseases, Allegante et al. [30] concluded that the patient’s quality of life can be improved through self-management while influencing his behavior through dedicated models. Van Riel et al. [31] are of the opinion that self-management by the patient with chronic disease plays a key role in his care. However, the relationship between doctor and patient must be characterized by joint decision-making. At the same time, it is necessary to educate patients and health professionals with the support of digital tools.

Self-management of people with low back pain requires a change in basic assumptions in healthcare. Patients should be given advice or empowered to know when to consult for diagnostic evaluation and symptom relief [32]. Optimal physiotherapy management for individuals with a previously conservatively managed primary traumatic shoulder dislocation requires education, progressive exercise, and an optional return-to-sport component. Behavior change strategies should be incorporated during the intervention [33]. To improve the quality of care for patients diagnosed with osteoarthritis of the knee or hip, Van Doormaal et al. [34] developed an evidence-based guideline to be used in conjunction with a laborious implementation strategy.

With the support of these medical practices, we developed indicator PB6–Patient self-care and self-management design (see Table A3), which is used to evaluate medical activity (PS–B) design of patient-centered medical services.

#### 2.4.2. Practices for Provision of Medical Services

A new model in health systems that facilitates better chronic care is the Innovative Chronic Care Framework. It allows the evaluation of its impact on the development of health policies and the re-design of healthcare [35]. By improving coordination and communication between social and health services, integrated care models offer solutions to the fragmentation of care [36].

Smith [37] shows that shared decision-making must consider findings from current evidence-based practice, in addition to the patient’s values, wishes and preferences. Physiotherapists treating patients with varying degrees of impairment and activity limitations are aided in making decisions by clinical practice guidelines [38]. Nurse navigators have an essential role in regaining the confidence of patients with multiple chronic conditions who frequently miss hospital appointments [39].

Nichol et al. [40] show that the wider applicability of interactive digital interventions to support self-management in low back pain remains uncertain. There is a limited body of evidence supporting the improvement of care for patients with multiple chronic conditions with digitally supported interventions [41].

With the support of these medical practices, we developed indicator IA6–Critical features for improving surveillance of chronically ill patients (see Table A5), which is used to evaluate medical activity (IS–A) medical services delivery.

Some problems such as comorbidities, time constraints, and the emotional state of the patient and their companions complicate discharge. The reduction of readmission rates can be achieved with the support of a structured discharge process, as revealed by Luther et al. [42].

The reduction of adverse events, as well as the improvement of the discharge process, is achieved through effective planning and communication [43]. Education for the improvement of discharged patients shapes the skills and attitudes of health personnel. However, there was no improvement in the health outcomes of the patients [44]. Educational interventions have effects on patients’ emotional state after discharge, knowledge, and medication adherence. A discharge plan adapted to each patient in combination with post-discharge services at the interface between the hospital and the patient’s home reduces readmission rates. It can improve health outcomes without increasing costs [45]. 

Patients with discharge planning register lower costs of laboratory services, as indicated by Emes et al. [46]. Patients treated in hospital-at-home interventions have a lower readmission rate [47].

With the support of these medical practices, we developed indicator IB6–Interventions to reduce problems in discharged patients (see Table A7), which is used to evaluate medical activity (IS–B) patient transfer.

#### 2.4.3. Practices for Evaluation of Medical Services

The adoption of patient-centered care can be achieved by clinicians if they address three essential elements: partnership, communication, and health promotion [48]. The medical staff active in orthopedic surgery prefer studies conducted by opinion leaders to randomized controlled trials [49]. Flodgren et al. [50] show that local opinion leaders can be effective in promoting evidence-based practice. They can use a series of interventions such as involvement in didactic programs, sending educational materials, contacts with colleagues, organizing community meetings, and awareness visits [51].

Zhang et al. [52] designed a patient-specific seizure onset and termination detection algorithm based on closed-loop machine learning that is energy efficient. Zhang and Szolovits [53] developed patient-specific real-time alarm algorithms based on individual patients’ vital signs.

Stephen et al. [54] highlighted a need and an opportunity to improve the functional and reported outcomes of patients who underwent lower extremity joint arthroplasty.

With the support of these medical practices, we developed indicator EA6–Management of patient-specific matters (see Table A9), which is used to evaluate medical activity (ES–A) institutional outlook leaders.

Some studies suggest an easier recovery of patients who have higher satisfaction levels [55]. Thus, it can be considered that the effectiveness of medical interventions impacts the patient’s level of satisfaction. The most important factors of patient satisfaction are communication, information, and respect for human dignity [56,57]. 

In evaluating a medical service, a particularly important aspect is the direct relationship between the medical staff and the patient. Along with this, the availability of a doctor, medical care, conditions in the ward, and the patient’s involvement in the treatment process, all offer clear information as well as an improved state of health [58]. Preoperative diagnosis, location of surgery, and length of stay do not significantly affect hospital or clinician satisfaction [59]. Quality care requires concise and time-efficient communication between the multidisciplinary medical team, patient, and family, as revealed in the study of Mercedes et al. [60].

Rosenblum et al. [61] studied the relationship between strategic and operational management of patient satisfaction in several hospitals in the USA, UK, Israel and Denmark. The managers of these hospitals state that it is a priority to know the satisfaction of the patient, but they do not involve the clinicians in this process, and they do not have a structured plan for improvement.

With the support of these medical practices, we developed indicator EB6–Patient satisfaction with therapeutic benefits (see Table A11), which is used to evaluate medical activity (ES–B) satisfaction degree evaluation.

#### 2.4.4. Practices for Medical Services Improvement

Issues affecting patient safety can be identified through detailed analysis of patient complaints. In this field, it is necessary to standardize the way in which patients’ complaints are analyzed and interpreted. Reader et al. [62] propose a coding taxonomy in the analysis of these data. Greiner et al. [63] assigned each patient a complaint code. Coded documentation of complaints allows for symptom-based analysis of care provided in emergency departments. Research conducted by complaint review boards or other internal hospital mechanisms should increase the fairness of medical processes and address the evolving demands of patients [64]. 

Gyberg et al. [65] show that patients and relatives play an active role in patient health and safety. Their needs include navigating the healthcare organization, understanding themselves and what is happening, and recognizing needs.

More than half of patients diagnosed with cancer reported not receiving adequate information, not being listened to, and being treated with disrespect or impersonality [66]. The use of structured communication strategies has the effect of improving communication and reducing the number of complaints in the outpatient setting [67].

With the support of these medical practices, we developed indicator RA6–Complaints management (see Table A13), which is used to evaluate medical activity (RS–A) self-appraisal.

The scientific literature shows several effective outcomes related to incident reporting that have led to improved staff communication regarding patient safety, reduced adverse drug events, increased one-year survival rates, decreased mortality, and improved compliance with work processes and medical care for monitoring patients’ condition [68].

Patient safety requires optimization that can be ensured by designing a learning system with inputs to identify obstacles and opportunities and implement plans to achieve success under the coordination of health care professionals [69]. Double-loop learning involves the use of adaptive management that can reduce uncertainty about processes that influence resource dynamics, as well as decision-making elements related to incidents. In order for corrective actions to be implemented in a timely manner through effective reporting, analysis, and investigation, the safety feedback loop must be closed. They must effectively address vulnerabilities in existing work systems [70].

To increase the effectiveness of incident reporting, local and national systems must work in close cooperation. This facilitates the transfer of good practices within an organization and between organizations [71]. Härkänen et al. [72] show that inadequate staffing levels, workload, and rush activity can lead to patient injury because of various omissions and errors. Consequently, incident reports should be analyzed in real time with the support of digital text analysis systems. These can monitor and flag medication errors as well as inadequate levels of staffing.

With the support of these medical practices, we developed indicator RB6–Incident report (see Table A15), which is used to evaluate medical activity (RS–B) medical services re-conception.

### 2.5. Format and Evaluation of Indicators

Having as input elements the descriptions of patient matters from the previous sections, the format and the contents of indicators was designed in the next stage of the research. We first developed the contents of the indicators, and subsequently designed the evaluation method.

Starting from the indicators’ contents, we developed evaluation questions to cover the aspects described by the indicators (see Table A1, Table A3, Table A5, Table A7, Table A9, Table A11, Table A13 and Table A15 for the patient matters indicators). Then we designed a grid for evaluating the degree of fulfillment of each indicator. They contain six steps described numerically (in interval 0–5) and qualitatively (a textual description of the respective level of achievement of the indicator, from not relevant to excellent), as follows: 0 (not relevant), 1 (low), 2 (satisfactory), 3 (good), 4 (very good), 5 (excellent) (see Table A2, Table A4, Table A6, Table A8, Table A10, Table A12, Table A14 and Table A16 for a detailed description of the evaluation grids for the fulfillment degree of the patient matters indicators).

To ensure that the evaluation presents a high degree of accuracy, we have associated a variable of importance with each indicator. They also contain five steps, which are described numerically and qualitatively (the textual description of the respective level of importance of the indicator), as follows: 0 (not relevant), 1 (unimportant), 2 (reduced importance), 3 (important), 4 (very important), 5 (high importance) [19,73]. The textual description of the respective level of importance of the indicator was elaborated according to how the failure to fulfill the indicator can compromise activity within the health unit, as can be seen in Table 1 [19,73].

Considering the extensive content of the eight indicators that describe patient issues and the related evaluation grids, we presented them in Table A1, Table A2, Table A3, Table A4, Table A5, Table A6, Table A7, Table A8, Table A9, Table A10, Table A11, Table A12, Table A13, Table A14, Table A15 and Table A16 of Appendix A, as follows: Table A1. Indicator PA6–Information regarding performance; Table A2. Scale for indicator PA6–Information regarding performance; Table A3. Indicator PB6–Patient self-care and self-management design; Table A4. Scale for indicator PB6–Patient self-care and self-management design; Table A5. Indicator IA6–Critical features for improving surveillance of chronically ill patients; Table A6. Scale for indicator IA6–Critical features for improving surveillance of chronically ill patients; Table A7. Indicator IB6–Interventions to reduce problems in discharged patients; Table A8. Scale for indicator IB6–Interventions to reduce problems in discharged patients; Table A9. Indicator EA6–Management of patient-specific matters; Table A10. Scale for indicator EA6–Management of patient-specific matters; Table A11. Indicator EB6–Patient satisfaction with therapeutic benefits; Table A12. Scale for indicator EB6–Patient satisfaction with therapeutic benefits; Table A13. Indicator RA6–Complaints management; Table A14. Scale for indicator RA6–Complaints management; Table A15. Indicator RB6–Incident report; Table A16. Scale for indicator RB6–Incident report.

For example, this is the way in which indicator PA6–Information regarding performance is defined in Table A1: by using performance indicators and publishing data on performance in the quality of medical care, it is expected that patients and clients can better orient themselves in selecting the desired healthcare services, and organizations and healthcare professionals can better decide what to offer, improve or purchase. The questions formulated for its evaluation are: Are performance indicators used to evaluate medical services? What are these? Are health facility performance data on quality of care published? Is there an assessment of patient and client orientation in the selection of healthcare services following the publication of healthcare facility performance data? Did the evaluation using performance indicators lead to service improvements? Have new infrastructure purchases been made with direct effects on the services offered? 

The evaluation scale of indicator PA6–Information regarding performance, presented in Table A2, consists of the following scores: 1–Low: Medical services are evaluated using performance indicators; 2–Satisfactory: Evaluation with the support of performance indicators led to improvements in health care services; 3–Good: Data related to the performance of the healthcare facility on the quality of medical care are published periodically and are accessible to the public; 4–Very good: Following the publication of data related to the healthcare facility’s performance, the orientation of patients and clients in the selection of healthcare services is evaluated, and the data is analyzed by the management of the organization; 5–Excellent: To improve the technical quality of the medical services offered, based on the results of the management analyses, new purchases of medical infrastructure/equipment were made with direct effects on the services offered.

To validate the developed theoretical model in practice, in the continuation of the research we conducted testing at the County Emergency Clinical Hospital Targu Mures, within the Orthopedics Department (CECHM) [74].

**Figure 2:**
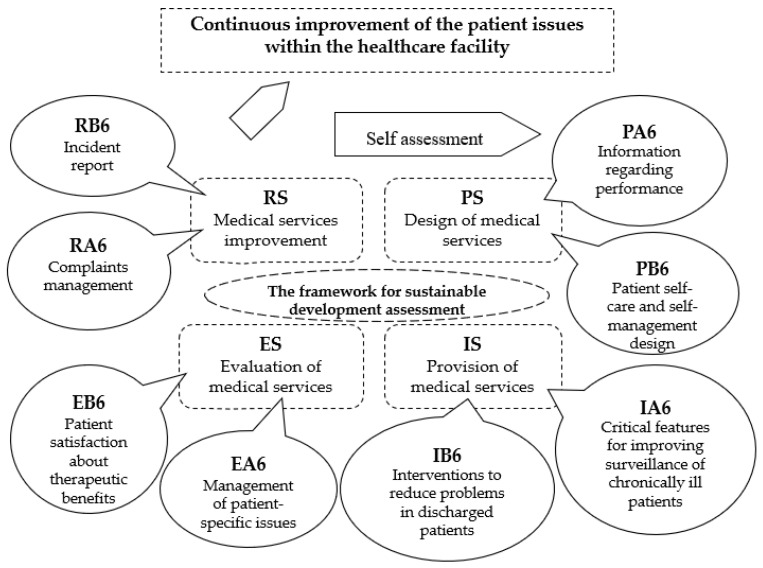
The patient matters continuous improvement cycle within the healthcare facility.

For this, we followed the sequence of indicators from the continuous improvement cycle, which is presented in Figure 2. The grids of these indicators for evaluating patient matters are presented in Table A1, Table A2, Table A3, Table A4, Table A5, Table A6, Table A7, Table A8, Table A9, Table A10, Table A11, Table A12, Table A13, Table A14, Table A15 and Table A16. Corresponding to the first cycle planning stage, we evaluated indicators PA6–Information regarding performance and PB6–Patient self-care and self-management design. In the implementation phase we used indicators IA6–Critical features for improving surveillance of chronically ill patients and IB6–Interventions to reduce problems in discharged patients. The third phase was carried out with indicators EA6–Management of patient-specific matters and EB6–Patient satisfaction with therapeutic benefits. Finally, for the review we employed indicators RA6–Complaints management and RB6–Incident report.

## 3. Results

**Figure 3:**
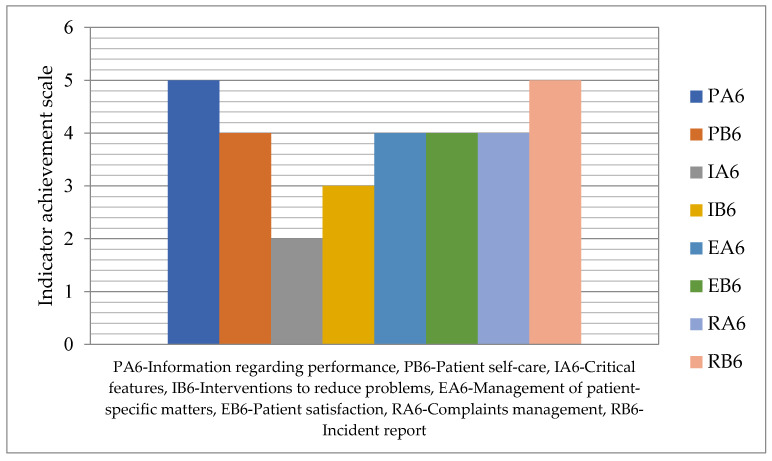
The degree of achievement of indicators related to patient matters.

**Figure 4:**
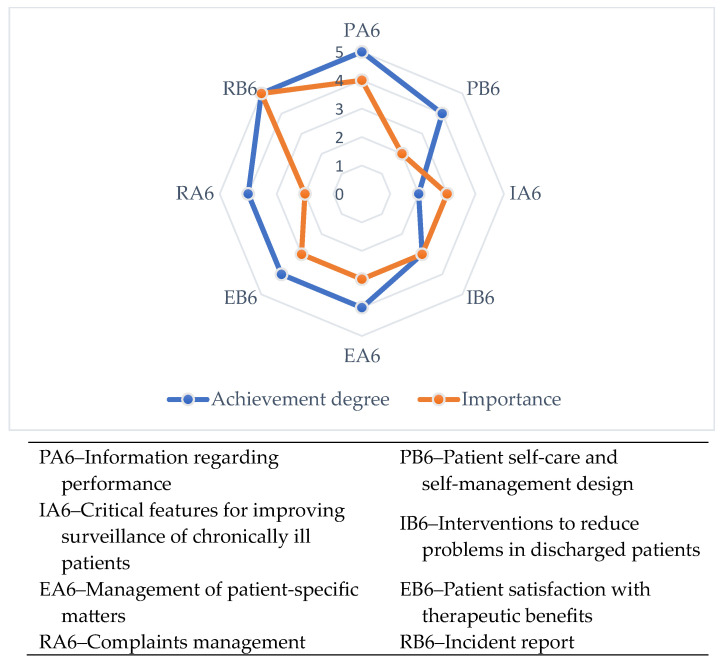
The patient matters evaluation graph.

**Figure 5:**
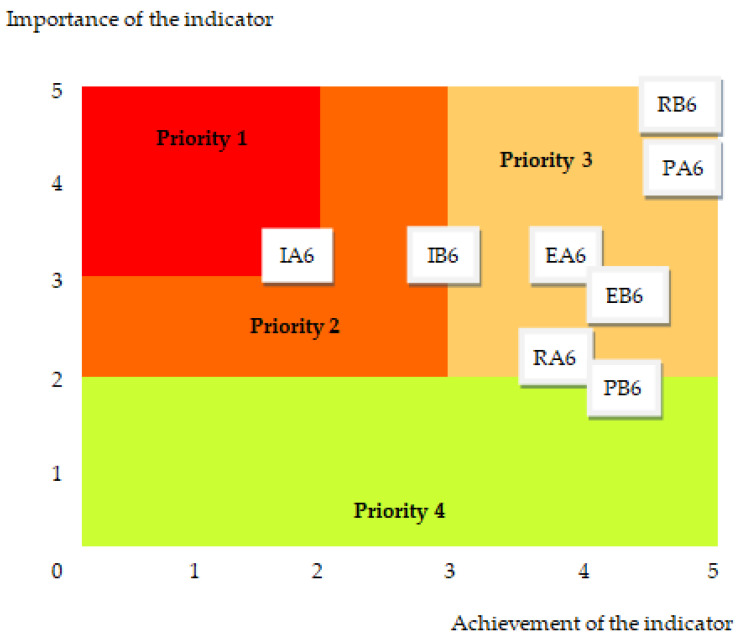
The assessment diagram for patient matters: PA6–Information regarding performance, PB6–Patient self-care and self-management design, IA6–Critical features for improving surveillance of chronically ill patients, IB6–Interventions to reduce problems in discharged patients, EA6–Management of patient-specific matters, EB6–Patient satisfaction with therapeutic benefits, RA6–Complaints management, RB6–Incident report.

The results of this study allowed:

- Elaboration of a patient matters indicators matrix that integrates the designed indicators in relation to the basic medical activities with the stages of the quality cycle (see Table 2, Table A1, Table A2, Table A3, Table A4, Table A5, Table A6, Table A7, Table A8, Table A9, Table A10, Table A11, Table A12, Table A13, Table A14, Table A15 and Table A16);
- Presentation of the eight indicator evaluation results (PA6–RB6) at an emergency hospital;
- Elaboration of the format and calculation of the values of the patient matters responsibility indicators: the global sustainability indicator for patient matters in current value and maximum value (Equations (1) and (2)), the overall patient matters sustainability level (Equation (3));
- Development of graphic tools for the analysis and assessment of responsibility regarding patient matters with the support of which improvement measures are identified and prioritized: self-assessment tool (Table 3), the degree of achievement of indicators (Figure 3), evaluation graph (Figure 4), assessment diagram (Figure 5).

The details of these results are presented in this section. With the support of medical practices related to patient matters (see Section 2.4), we projected the indicator matrix associated with the patient matters responsibility of the Health-Sustainability reference framework (Table 2).

From this analysis, one can see the connection between the eight basic medical activities of the quality cycle (column 2) and social responsibility for patient matters (column 3). These connections were established due to discovery in the scientific literature of some links between basic medical activities and social responsibility for patient matters. The connections are also reflected in the names given to the indicators. 

We tested the validity and reliability of the newly created assessment tool by distributing it to four experts in the assessment of the quality of medical care services within CECHM. They tested the intelligibility, readability, and completeness of the description of the content of the indicators, the questions for evaluation, the levels that describe the degree of fulfillment of the indicators, and the criteria by which the importance levels of the indicators were established. The results allowed for improvements in the contents and format of editing the indicators, support for collecting and processing information, and also some reframing in the indicators’ assessment grids. The data collected in this test were used in the final formulation of the assessment tool content. The tool was recognized as a reliable and validated measure for the comprehensive assessment of patient matters.

In previous research, indicator matrices along with indicator contents are introduced in the following areas of social responsibility: human rights [19], labor practices [75], environment [73], and fair healthcare practices [16]. This research details the patient issues area of social responsibility and the eight indicators that make up its composition. The following section presents the results and findings of our study following the evaluation of patient matters indicators at an emergency hospital.

PA6–Information regarding performance—Hospital activity is continuously monitored through the performance indicators of the manager, as well as those included in the ongoing contracts with the Mures Health Insurance Company. The quantitative indicators used are the number of discharges for continuous and day hospitalization. The qualitative indicators are the case mix index (CMI) or the case complexity index at ward and hospital level. The projected CMI for 2022 was 1.4995, and the achieved CMI had higher values in each month of 2022, from 1.9984 (January) to 2.5125 (December).

The management performance indicators adopted by the management contract are: (1) Indicators of service use: Average length of hospitalization within the hospital and on each ward (the value of the indicator assumed by the contract was 7.62—the achieved value of the indicator was 6.78—degree of achievement 89%); Bed utilization rate within the hospital and on each ward (74.14–43.91–59%); The index of complexity of cases within the hospital and on each ward (1.4995–2.1745–145%); The percentage of patients undergoing surgical operations within the total number of patients discharged from surgical wards (70–75.09–107%); (2) Quality indicators: In-hospital mortality rate, in the whole hospital and on each ward (11.27–6.62–59%); The rate of nosocomial infections, in the whole hospital and on the wards (4.40–1.85–42%); Concordance index between admission and discharge diagnoses (59.36–70.75–119%).

The efficiency indicators of the departments and compartments with beds, for example the Orthopedics and Traumatology Clinical Department, are: number of provided beds—70; average number of beds—70; number of hospitalized patients—1544; bed utilization index—137.11; average duration of hospitalization—6.22; hospital mortality—1.76; turnover of patients—22.06; bed utilization rate—37.46.

To improve the technical quality of the medical services offered, in the course of 2022 modern high-performance medical equipment worth 217,799 euros was purchased, which had direct effects on the services offered.

PB6–Patient self-care and self-management design—The medical staff provide information according to the needs of each patient, starting from the finding that level of knowledge influences behavior. In this self-care design model, decisions about the therapeutic plan belong to the doctor, and the patient is asked to adhere to this plan. There is concern for establishing ways of communicating with the patient to achieve partnership and make collaborative clinical decisions. The medical team provides support for self-care through the support measures they provide to patients who are in a training process. As a result, patients acquire intervention skills supported by behavior modification techniques and adherence to the treatment plan. Self-management of patients is designed through the transmission of medical information: leaflets, magazines, web resources, etc.; understanding the model of assistance in building self-care skills; ongoing support from the healthcare team, family and self-help groups in the community.

IA6–Critical features for improving surveillance of chronically ill patients—Patients with chronic conditions are given information about diagnosis, treatment options and access to specialist care, and the right to make their own decisions. In the case of palliative care, when the patient no longer has decision-making capacity, the information and decision are transferred to the family or staff providing palliative care. Care is patient-centered given that the perception of suffering is subjective. Advice is provided on symptom control, pain management, holistic care (medical, social, psycho-emotional), and psychosocial support. In the orthopedic clinic, robotic technologies [76,77], computer technologies [78,79], and mechatronic rehabilitation systems [80] are used.

IB6–Interventions to reduce problems in discharged patients—During hospitalization, an extensive geriatric assessment is conducted, which aims at the medical and psycho-social assessment of the patient. Upon discharge, the attending physician assesses the patient’s state of health and issues a medical letter to the family doctor or specialist in the outpatient clinic which gives indications for treatment and therapeutic supervision for the next period. After discharge, the family doctor monitors the patient’s health status, in certain cases through the telemedicine system.

EA6–Management of patient-specific matters—Local opinion leaders invite recognized experts in the field who make specialized presentations to the ward staff regarding patient-specific therapies, good medical practices, and monitoring their effective implementation. Access to national curative health programs and priority actions for conditions, as well as national public health programs, is promoted (e.g., RT-PCR testing for 2019-nCOV for 29,020 patients).

EB6–Patient satisfaction with therapeutic benefits—In order to evaluate the therapeutic benefits of the medical services provided during hospitalization, in 2022, the health services quality management service processed 1519 questionnaires that were completed by patients. The communication of doctors with patients was evaluated (73.60%—very satisfied; 20.01%—satisfied; 5.46%—no answer; 0.92%—unsatisfied), the care provided by doctors (75.05%—very satisfied; 19.03%—satisfied; 5.46%—no answer; 0.46%—dissatisfied), the communication of nurses with patients (74.39%—very satisfied; 20.34%—satisfied; 4.94%—no answer; 0.33%—dissatisfied), the nursing activity of nurses (73.14%—very satisfied; 20.47%—satisfied; 6.06%—no answer; 0.33%—dissatisfied), post-operative care including Anesthesia and Intensive Care (52.28%—very satisfied; 24.92%—satisfied; 22.27%—no answer; 0.53%—dissatisfied), if the patient will return to the hospital in case of needing a medical service (89.86%—yes; 7.44%—probably; 2.17%—no answer; 0.53%—no), etc. For each of the aspects studied, the evolution of the patient’s satisfaction degree is evaluated compared to the previous evaluations.

Patients admitted to the hospital reported both positive and negative aspects, and made proposals to improve medical services which were submitted to the Steering Committee. 

RA6–Complaints management—The number of patient complaints that were registered during the year 2022 is five. Compared to the value of the indicator assumed by the management contract, which is 150 complaints, this means a degree of achievement of 5%. 

The level of satisfaction of staff making complaints is monitored by the hospital’s Ethics Board. During the year 2022, only one complaint was registered, and that was resolved by staff training provided by the head of the department using the hospital’s Internal Regulations and the Code of Conduct for contractual staff within the hospital.

RB6–Incident report—According to the system procedure for reporting, analysis and monitoring of adverse events, sentinel and near-miss, the health unit reports without apportioning blame through the CAPESARO application to the National Authority for Quality Management in Healthcare (NAQMH) any adverse events associated with medical care, in order to learn from mistakes. The event is analyzed by a committee that presents a report to the hospital manager. The quality management service presents to the NAQMH a report analyzing the causes of the event and the proposed measures to avoid its recurrence. During 2022, 262 adverse events associated with healthcare were reported, a decrease of 35.62% compared to 2021, when 407 events were reported.

The values achieved for the indicators related to patient matters responsibility are registered in the self-assessment tool (Table 3).

The degree of achievement of indicators related to patient matters is depicted in Figure 2 on a scale in the range 1–5.

In this domain, indicator IA6–Critical features for improving surveillance of chronically ill patients has a minimum value of 2, while the highest value of 5 is recorded for indicators PA6–Information regarding performance and RB6– Incident report.

The radar-type evaluation graph in Figure 3 highlights two characteristics of indicators related to patient matters: achievement degree and importance.

The sum of individual sustainability indicators from Table 2 reflects the global sustainability indicator for patient matters (GS_PM_):

(1) $${\mathrm{GS}}_{\mathrm{PM}}=\overset{8}{\underset{i=1}{\sum }}S_{i}=\overset{8}{\underset{i=1}{\sum }}I_{i}\cdot A_{i}=100$$

The maximum value of global sustainability for patient issues is the sum of the maximum values of the indicators that compose it (GSmax_PI_):

(2) $${\mathrm{GSmax}}_{\mathrm{PM}}=5\cdot \overset{8}{\underset{i=1}{\sum }}I_{i}=5\cdot 25=125$$

By reporting the percentage of the obtained values, we can calculate the overall patient matters sustainability level (LGS_PM_):

(3) $${\mathrm{LGS}}_{\mathrm{PM}}=\frac{{\mathrm{GS}}_{\mathrm{PM}}}{{\mathrm{GSmax}}_{\mathrm{PM}}}\cdot 100=\frac{100}{125}\cdot 100=80\%$$

This result reflects the degree to which the hospital meets the requirements regarding patient matters. Furthermore, we have represented the couples of values related to the 8 indicators in a prioritization framework of the Eisenhower matrix type (Figure 5). It allows identification of the degree of importance and urgency of subsequent actions, from high priority (1) up to low priority (4).

For this evaluation to improve patient matters, highest priority should be given to indicator IA6–Critical features for improving surveillance of chronically ill patients.

## 4. Discussion

In the practical implementation stage, we validated responsibility regarding patient matters using the components of the innovative H-S reference framework at the CECHM emergency hospital. The team of evaluators included medical personnel and specialists in quality assurance: the chief physician, orthopedic doctor, and chief assistant responsible for quality assurance.

A first finding of the study was the adequacy of the content of the indicators designed for the evaluation of patient issue responsibility with the proposed purpose. They are in accordance with the requirements of medical practices in international hospitals. We found the projected indicators compatible with the European DUQuE hospital quality assessment framework [13], as well as with the national accreditation legislation for sanitary units with beds [14], and with the accreditation requirements for outpatient health services [15]. In contrast to these, the frame of reference elaborated in this research has the added value of directing the medical staff, patients and interested parties towards sustainability.

However, in some situations it was necessary to adjust the content of the indicators to the specifics of CECHM. From here came a first recommendation, that before evaluation the auditors should analyze the content of the indicators and adapt them as best they can to the realities of the evaluated healthcare facility. The development of a glossary of terms would facilitate a good understanding of the notions used by all parties involved in the evaluation.

Another finding was related to the planning of the evaluation audit. It is necessary to have effective communication with the representatives of the audited departments so that they reserve the time necessary for the audit and provide the necessary evidence. For this reason, it is recommended that the leader of the team of evaluators be a person with authority, and a good planner and organizer.

Through the pilot implementation of the new frame of reference and the evaluation of the developed indicators, we have found that we have contributed to increasing the responsibility of health personnel regarding patient matters. Thus, we created the framework for a complex evaluation of medical and administrative practices related to patient matters which makes a direct contribution to increasing the sustainability of hospital processes.

The results of our study indicate that indicator IA6–Critical features for improving surveillance of chronically ill patients should be addressed as a priority. For its improvement, it is necessary that the surveillance system for patients with chronic conditions (e.g., sacroiliac arthritis) ensures decision support at the time and place of decision making. Also, this system, based on the periodic assessment of patients, must provide them with recommendations. Decision-making support for improving the supervision of patients must use new IT technologies and be generated with the help of a computer. At the same time, there is a need for additional training of the medical staff regarding the correct use of digital surveillance technologies.

As concluded by Torrens et al. [49], we found that in orthopedic surgery the medical community pays more attention to opinion leader studies than to randomized controlled trials. Local opinion leaders are effective in promoting evidence-based practice, participating in teaching programs, distributing educational materials, and regularly organize meetings with colleagues.

Like the findings of the study conducted by Boudreau et al. [6], we found that patients overestimate the level of reimbursement of the orthopedic surgeon by the Health Insurance Company. For this reason, better transparency is needed to increase patients’ trust.

Contrary to Schöni and Waibel [8], we found that in the hospital, through the orthopedic technique used, the specific complications of patients with chronic conditions, such as chronic diabetic foot wounds, are treated accordingly.

Like the findings of the study conducted by Van Riel et al. [31], we found that patients with chronic diseases (e.g., coxarthrosis, gonarthrosis) who self-manage have better therapeutic results, because the importance they give to medical care increases. In general, the applied care model is based on a doctor-patient relationship in which they make joint decisions. On the other hand, we did not find the existence of “hospital at home” type interventions through which the treated patients have a lower hospitalization rate, as indicated in the study by Mas et al. [47].

In agreement with the results reported by Asfuroğlu and Gümüşoğlu [10], we found that following the COVID-19 pandemic, the outpatient clinic saw a decrease in the number of patients with minor orthopedic conditions such as contusions, sprains, etc. which can be treated by the family doctor. There was also an increase in the number of severe orthopedic cases (e.g., acetabular fractures, hip fractures, etc.) of patients from other regions of the country, who have good confidence in the surgical interventions at CECHM.

Unlike Mitchell et al. [11], who claim that incident reports do not reach their potential, we found at CECHM that they are properly processed in terms of triage, analysis and recommendations addressed to patients, and doctors have an adequate level of involvement.

The small number of five complaints formulated during the year 2022 registered at CECHM leads us to appreciate that our study has findings contrary to the results reported by Friele et al. [12]. They are of the opinion that patients who formulate complaints have negative public preconceptions about how the complaint is treated. Our analysis did not highlight opposing expectations of patients and doctors in relation to the subject of complaint. 

## 5. Limitations

The study we conducted has some limitations. The development of the indicators of the new reference framework based on the successful practices described in the scientific literature may omit certain aspects that can be encountered in some healthcare facilities. It is possible that certain requirements of the healthcare facilities have not been addressed, and the use of the new reference framework will require a careful analysis of the indicators and an adaptation to the evaluated medical specialties, to the characteristics of the hospital in terms of organizational form and ownership form, and also of the particular conditions in which it operates. Another limitation of the study is due to the practical validation of the reference framework at an orthopedic hospital. The evaluation of other medical specialties could lead to the augmentation of indicator content with specific aspects, to increase the area of medical fields covered. From these limitations come future directions of study to diversify the content of the indicators so that they respond to the most branched medical activities. Another direction of study consists of the digitization of the evaluation process with the support of IT tools, which would allow good traceability and management of continuous improvement programs.

## 6. Conclusions

In this study, we presented the aspects of social responsibility that describe patient matters. Their evaluation within healthcare facilities is conducted with the support of eight indicators that make up the new health-sustainability reference framework. The design of the indicators based on medical practices reported in the scientific literature allowed us to confirm the adequacy of their contents with the proposed purpose. The description of the indicators, and the means of evaluation through the importance-degree of achievement couple on scales with values from 0 to 5, constitutes an innovative element of the research. With this support, the performance level of the healthcare facility can be evaluated in terms of responsibility for patient matters. The representation of the indicators in an Eisenhower matrix prioritization framework allows the development of improvement measures according to their degree of importance and urgency.

The practical validation of the research results highlighted the compatibility of the new reference framework with the European framework for quality assurance in hospitals, with the national legislation for the accreditation of healthcare facilities with beds, and with the national legislation for the accreditation of outpatient facilities. The added value in relation to these references consists in promoting the sustainable development of the healthcare facility, as well as the awareness and orientation of the health staff and patients towards sustainability.

## Figures and Tables

**Table 1:** Importance of indicators [19,73].

Value [I]	Importance Category	Description
0	Not relevant	X
1	Unimportant	The assessed requirement is of little importance for the healthcare facility and there is a marginal tendency for evaluation.
2	Reduced importance	Failure to comply with this requirement could adversely affect the activity of the healthcare facility.
3	Important	Failure to comply with the requirement could compromise the activity of the healthcare facility. Compliance is essential to meet the requirements of the healthcare facility.
4	Very important	Failure to meet this requirement could jeopardize the successful provision of healthcare. Fulfilling the requirement is essential for the successful delivery of healthcare.
5	High importance	Failure to comply with the requirement may even compromise the existence of the healthcare facility.

**Table 2:** Patient matters indicator matrix of the H-S framework.

Basic Medical Activities in Quality Cycle		Patient Matters—Social Responsibility
(PS) Design of medical services	(PS–A) Accreditation of health system	PA6–Performance information (Table A1 and Table A2)
	(PS–B) Design of patient-centered medical services	PB6–Patient self-care design and self-management (Table A3 and Table A4)
(IS) Provision of medical services	(IS–A) Medical services delivery	IA6–Critical features for improving the surveillance of patients with chronic conditions (Table A5 and Table A6)
	(IS–B) Patient transfer	IB6–Interventions to reduce problems in outpatients (Table A7 and Table A8)
(ES) Evaluation of medical services	(ES–A) Institutional outlook leaders	EA6–Patient-specific issues management (Table A9 and Table A10)
	(ES–B) Satisfaction degree evaluation	EB6–Patient satisfaction degree regarding therapeutic benefits (Table A11 and Table A12)
(RS) Medical services improvement	(RS–A) Self-appraisal	RA6–Complaints management (Table A13 and Table A14)
	(RS–B) Medical services re-conception	RB6–Incident report (Table A15 and Table A16)

**Table 3:** Self-assessment tool for patient matters responsibility.

No.	Indicator Description	Importance (Ii)	Achievement (Ai)	Sustainability Indicator (Si = Ii·Ai)
1	PA6–Information regarding performance	4	5	20
2	PB6–Patient self-care and self-management design	2	4	8
3	IA6–Critical features for improving surveillance of chronically ill patients	3	2	6
4	IB6–Interventions to reduce problems in discharged patients	3	3	9
5	EA6–Management of patient-specific matters	3	4	12
6	EB6–Patient satisfaction with therapeutic benefits	3	4	12
7	RA6–Complaints management	2	4	8
8	RB6– Incident report	5	5	25

Ii—Importance, Ai—Achievement, Si—Sustainability Indicator.

## Data Availability

The data used in this study can be requested from the corresponding author.

## Appendix A

**Table A1:** Indicator PA6–Information regarding performance.

Indicator	PA6–Information Regarding Performance
Description	By using performance indicators and publishing data on performance in the quality of medical care, it is expected that: – Patients and clients can better orient themselves in selecting the desired healthcare services; – Organizations and healthcare professionals can better decide what to offer, improve or purchase.
Evaluation question	Are performance indicators used to evaluate medical services? What are these? Are health facility performance data on quality of care published? Is there an assessment of patient and client orientation in the selection of healthcare services following the publication of healthcare facility performance data? Did the evaluation using performance indicators lead to service improvements? Have new infrastructure purchases been made with direct effects on the services offered?

**Table A2:** Scale for indicator PA6–Information regarding performance.

Score [A]	Achievement	Content
0	Not relevant	–
1	Low	Medical services are evaluated using performance indicators.
2	Satisfactory	Evaluation with the support of performance indicators led to improvements in health care services.
3	Good	Data related to the performance of the healthcare facility on the quality of medical care are published periodically and are accessible to the public.
4	Very good	Following the publication of data related to the healthcare facility’s performance, the orientation of patients and clients in the selection of healthcare services is evaluated, and the data is analyzed by the management of the organization.
5	Excellent	To improve the technical quality of the medical services offered, based on the results of the management analyses, new purchases of medical infrastructure/equipment were made with direct effects on the services offered.

**Table A3:** Indicator PB6–Patient self-care and self-management design.

Indicator	PB6–Patient Self-Care and Self-Management Design
Description	Designing patient-supported interventions: – self-help groups and peer support to improve self-care and self-management [30]; – patient involvement in prevention (e.g. infection control) to improve patient safety [33]; – theories or models are used that consider a wide range of influences on behavior.
Evaluation questions	Are patient-supported interventions designed? What are the patient-supported interventions? Are there self-help groups and patient peer support? Do patients apply self-care and self-management? Are patients involved in preventive actions that increase their safety? Is patient behavior influenced by theories or models?

**Table A4:** Scale for indicator PB6–Patient self-care and self-management design.

Score [A]	Achievement	Content
0	Not relevant	–
1	Low	Depending on their capabilities and wishes, patients are supported and given the opportunity to participate in their own care.
2	Satisfactory	Healthcare decision-making is the result of a shared process at the interface between the patient and the medical team. This relates to mediated treatment or prevention decisions as well as long-term planning for complex needs.
3	Good	Patients organize themselves into self-help and peer support groups. Behavior change strategies are incorporated.
4	Very good	Patients apply self-care and self-management. Patients with chronic diseases manage their condition most of the time. This is supported by theories or models that account for a wide range of influences on behavior.
5	Excellent	Patients participate in actions to prevent medical suffering, which increase their safety. There is a doctor-patient relationship in which they make decisions together.

**Table A5:** Indicator IA6–Critical features for improving surveillance of chronically ill patients.

Indicator	IA6–Critical Features for Improving Surveillance of Chronically Ill Patients
Description	To improve clinical practice, critical features of the surveillance system for patients with chronic conditions must include: – automated provision of decision support as part of clinicians’ workflow [37]; – ensuring decision-making support at the time and place of decision-making [38]; – providing a recommendation and not just an assessment [39]; – using a computer to generate decision support [40].
Evaluation questions	Does the chronic patient surveillance system automatically provide decision support as part of clinicians’ workflow? Is decision support provided at the time and place of decision making? Are recommendations provided to patients during assessments? Is a computer used in the generation of decision support?

**Table A6:** Scale for indicator IA6–Critical features for improving surveillance of chronically ill patients.

Score [A]	Achievement	Content
0	Not relevant	–
1	Low	A goal of the healthcare facility is to improve clinical practice, with the support of the critical features of the surveillance system for patients with chronic conditions.
2	Satisfactory	The chronic pain surveillance system automatically provides decision support as part of clinicians’ workflow.
3	Good	The surveillance system for chronically ill patients provides decision-making support at the time and place of decision-making.
4	Very good	The surveillance system for patients with chronic conditions provides not only evaluations but also recommendations.
5	Excellent	To improve the supervision of patients with chronic conditions, decision support is generated with the support of a computer.

**Table A7:** Indicator IB6–Interventions to reduce problems in discharged patients.

Indicator	IB6–Interventions to Reduce Problems in Discharged Patients
Description	Activities aimed at reducing problems in adult patients discharged from hospital [45]: – pre-admission assessment; – complete discharge planning protocols; – extensive geriatric assessment; – support arrangements for discharge; – educational activities.
Evaluation questions	Are pre-admission patient assessments conducted? Are discharge planning protocols complete? Is an extensive geriatric assessment performed? Are discharge support arrangements being made? Are staff and patient education activities used for discharge?

**Table A8:** Scale for indicator IB6–Interventions to reduce problems in discharged patients.

Score [A]	Achievement	Content
0	Not relevant	–
1	Low	Adult patients discharged from hospital were assessed at pre-admission.
2	Satisfactory	Patient discharge planning protocols are complete, and when possible, from the time of admission, an approximate date of discharge is communicated to the patient, which changes according to the clinical evolution.
3	Good	During the hospitalization, an extensive geriatric evaluation is conducted, which aims at the medical and psycho-social assessment of the patient, the diagnosis, and the development of strategies for prevention, treatment and rehabilitation.
4	Very good	Upon discharge, housing support arrangements are made, social support is available, the contact details of the person administering the treatment are known to the discharge healthcare facility and the public health office.
5	Excellent	The patient is informed about the monitoring program during the outpatient phase of treatment and given the hospital telephone number for advice. Discharge staff and patient education activities are conducted.

**Table A9:** Indicator EA6–Management of patient-specific matters.

Indicator	EA6–Management of Patient-Specific Matters
Description	Appropriate management of patient-specific matters occur with the support of local opinion leaders, who: – periodically send educational materials; – host a community meeting with a recognized expert in the field; – arrange awareness visits; – maintain and improve their regular formal and informal contacts with colleagues; – participate in didactic programs. Real-time alarm systems are used for critical patients. Systems are used that detect the onset and termination of patient-specific seizures.
Evaluation questions	Do local opinion leaders regularly send out educational materials? Do opinion leaders host community meetings with recognized experts in the field? Are there regular formal and informal contacts between opinion leaders and their peers? Are opinion leaders involved in teaching programs? Are real-time alarm systems used for critical patients? Are systems used that detect the onset and termination of patient-specific seizures?

**Table A10:** Scale for indicator EA6–Management of patient-specific matters.

Score [A]	Achievement	Content
0	Not relevant	–
1	Low	There are local opinion leaders who are recognized for their professionalism and inform themselves about the needs for improvement in the healthcare facility.
2	Satisfactory	Local opinion leaders periodically send educational materials to healthcare facility staff.
3	Good	Local opinion leaders regularly host healthcare facility staff meetings with recognized experts in the field.
4	Very good	Local opinion leaders maintain and improve their regular formal and informal contacts with colleagues. Critical patients are monitored with real-time alarm systems.
5	Excellent	Local opinion leaders are involved in teaching programs made available to the healthcare facility staff. Systems are used that detect the onset and termination of patient-specific seizures.

**Table A11:** Indicator EB6–Patient satisfaction with therapeutic benefits.

Indicator	EB6–Patient Satisfaction with Therapeutic Benefits
Description	Measure of patients’ satisfaction after completion of medical treatment, relative to their initial expectations of therapeutic benefits.
Evaluation questions	Is patient satisfaction measured after completion of medical treatment? Are the therapeutic benefits evaluated against their initial expectations?

**Table A12:** Scale for indicator EB6–Patient satisfaction with therapeutic benefits.

Score [A]	Achievement	Content
0	Not relevant	–
1	Low	There are updated questionnaires to assess patient satisfaction after completion of medical treatment, relative to their initial expectations of therapeutic benefits.
2	Satisfactory	Questionnaires assessing patients’ satisfaction after completion of medical treatment, in relation to their initial expectations regarding therapeutic benefits, are distributed periodically according to a procedure.
3	Good	The therapeutic benefits of medical treatment is evaluated in relation to the patients’ initial expectations.
4	Very good	Compared to the previous evaluation, the evolution of the patients’ degree of satisfaction regarding the therapeutic benefits of medical treatment is evaluated in relation to the patients’ initial expectations.
5	Excellent	Improvement measures are established to increase patient satisfaction after completion of medical treatment relative to their initial expectations of therapeutic benefits.

**Table A13:** Indicator RA6–Complaints management.

Indicator	RA6–Complaints Management
Description	Mechanisms/tools for identifying and resolving complaints.
Evaluation questions	Are there clear procedures for patients to make complaints against medical services? How are complaints managed? How many complaints were made against the organization last year? How many complaints were accepted as well-founded and how were they resolved? Are there improvements in medical services because of complaints made?

**Table A14:** Scale for indicator RA6–Complaints management.

Score [A]	Achievement	Content
0	Not relevant	–
1	Low	Complaints can be filed easily.
2	Satisfactory	There is a procedure for collecting and handling complaints.
3	Good	Complaints are managed according to the complaint collection and handling procedure.
4	Very good	Complaints accepted as well-founded have been resolved, and those who made them are informed of the outcome.
5	Excellent	Medical services have been improved because of the complaints made.

**Table A15:** Indicator RB6–Incident report.

Indicator	RB6–Incident Report
Description	Incident reporting, or root cause analysis, is an event analysis tool that allows retrospective analysis of systematic causes and prevents the recurrence of adverse events and errors that lead to death, serious physical or psychological injury, and the risk of such injury [13]. Feedback from incident reporting leads to improved patient safety in health care delivery [13,70]. The effectiveness of feedback from incident reporting systems is increased when the following aspects are incorporated/considered from the design stage [13,71]: – feedback is collected from several levels of the organization; – the suitability of the manner of presentation and transmission; – relevance of the content for the intended workplace and for the system; – integrating feedback into the design of safety information systems; – control of feedback and requirements of different users regarding the sensitivity of information; – empowering staff to assume responsibilities regarding safety improvement; – capability for rapid feedback cycles and immediate understanding of risks; – direct feedback to observers and key stakeholders; – feedback processes are established, clearly defined, continuous and understood in the same way by all participants; – integrating safety feedback into the work routine of frontline staff; – the improvements made are visible; – staff consider the source and content of the feedback to be credible; – feedback preserves confidentiality and increases trust between observers and policy makers [13]; – visible high-level support for initiatives to improve safety systems; – double-loop learning, by modifying objectives or decision-making rules because of accumulated experience.
Evaluation questions	Are incident reports developed that present the adverse events recorded in the organization, or errors that lead to death, serious physical or psychological harm, and the risk of such harm? At what levels are incident reports collected? Is the presentation and transmission of incident reports appropriate? Is the content of the incident reports relevant to the intended workplace and the system? Is feedback from incident reports integrated into the design of safety information systems? Is the feedback and requirements of different users on the sensitivity of the information controlled? Are staff empowered to take responsibility for improving safety? Is there capability for rapid feedback cycles and immediate understanding of risks? [71] Is feedback delivered directly to observers and key stakeholders? Are feedback processes established, clearly defined, ongoing and understood in the same way by all participants? Is safety feedback integrated into the work routine of frontline staff? Are improvements made through incident reports visible? Do staff find the source and content of the feedback credible? Does feedback provided through incident reports maintain confidentiality and increase trust between observers and policy makers? Do safety improvement initiatives enjoy visible high-level support? Is double-loop learning used, by changing the goals or decision rules because of experience?

**Table A16:** Scale for indicator RB6–Incident report.

Score [A]	Achievement	Content
0	Not relevant	–
1	Low	In the healthcare facility, incident reports are developed that present the adverse events recorded in the organization, or errors that lead to death, serious physical or psychological injuries, as well as the risk of such injuries.
2	Satisfactory	Incident reports are collected at all organizational levels. The presentation and submission of incident reports is appropriate, and the content of incident reports is relevant to the intended workplace and the system.
3	Good	There are rapid feedback cycles and immediate understanding of risks, and they are established, clearly defined, ongoing and understood in the same way by all participants. Feedback is provided through incident reports directly to observers and key stakeholders and is integrated into the design of safety information systems.
4	Very good	The staff believe that the source and content of the safety feedback is credible, and confidentiality is maintained, as well as the requirements of different users regarding the sensitivity of information. Staff are empowered to take responsibility for safety improvement, feedback is monitored, and it is integrated into the work routine of frontline staff.
5	Excellent	Improvements brought about by incident reports are visible, and initiatives to improve safety systems enjoy visible support from facility management. Double-loop learning is used by modifying the objectives or decision-making rules because of accumulated experience.
